# Acute respiratory failure in the elderly: etiology, emergency diagnosis and prognosis

**DOI:** 10.1186/cc4926

**Published:** 2006-05-24

**Authors:** Patrick Ray, Sophie Birolleau, Yannick Lefort, Marie-Hélène Becquemin, Catherine Beigelman, Richard Isnard, Antonio Teixeira, Martine Arthaud, Bruno Riou, Jacques Boddaert

**Affiliations:** 1 Department of Emergency Medicine and Surgery, Centre Hospitalo-Universitaire Pitié-Salpêtrière, Assistance Publique – Hôpitaux de Paris, Université Pierre et Marie Curie-Paris 6 75651 Paris Cedex 13, France; 2 Department of Pneumology and Respiratory Intensive Care Unit, Centre Hospitalo-Universitaire Pitié-Salpêtrière, Assistance Publique – Hôpitaux de Paris, Université Pierre et Marie Curie-Paris 6 75651 Paris Cedex 13, France; 3 Laboratory of Pulmonary Function Test and UPRES 2397, Centre Hospitalo-Universitaire Pitié-Salpêtrière, Assistance Publique – Hôpitaux de Paris, Université Pierre et Marie Curie-Paris 6 75651 Paris Cedex 13, France; 4 Department of Radiology, Centre Hospitalo-Universitaire Pitié-Salpêtrière, Assistance Publique – Hôpitaux de Paris, Université Pierre et Marie Curie-Paris 6 75651 Paris Cedex 13, France; 5 Department of Cardiology, Centre Hospitalo-Universitaire Pitié-Salpêtrière, Assistance Publique – Hôpitaux de Paris, Université Pierre et Marie Curie-Paris 6 75651 Paris Cedex 13, France; 6 Department of Internal Medicine, Centre Hospitalo-Universitaire Pitié-Salpêtrière, Assistance Publique – Hôpitaux de Paris, Université Pierre et Marie Curie-Paris 6 75651 Paris Cedex 13, France; 7 Laboratory of Emergency Biology, Centre Hospitalo-Universitaire Pitié-Salpêtrière, Assistance Publique – Hôpitaux de Paris, Université Pierre et Marie Curie-Paris 6 75651 Paris Cedex 13, France; 8 Department of Geriatry, Centre Hospitalo-Universitaire Pitié-Salpêtrière, Assistance Publique – Hôpitaux de Paris, Université Pierre et Marie Curie-Paris 6 75651 Paris Cedex 13, France

## Abstract

**Introduction:**

Our objectives were to determine the causes of acute respiratory failure (ARF) in elderly patients and to assess the accuracy of the initial diagnosis by the emergency physician, and that of the prognosis.

**Method:**

In this prospective observational study, patients were included if they were admitted to our emergency department, aged 65 years or more with dyspnea, and fulfilled at least one of the following criteria of ARF: respiratory rate at least 25 minute^-1^; arterial partial pressure of oxygen (PaO_2_) 70 mmHg or less, or peripheral oxygen saturation 92% or less in breathing room air; arterial partial pressure of CO_2 _(PaCO_2_) ≥ 45 mmHg, with pH ≤ 7.35. The final diagnoses were determined by an expert panel from the completed medical chart.

**Results:**

A total of 514 patients (aged (mean ± standard deviation) 80 ± 9 years) were included. The main causes of ARF were cardiogenic pulmonary edema (43%), community-acquired pneumonia (35%), acute exacerbation of chronic respiratory disease (32%), pulmonary embolism (18%), and acute asthma (3%); 47% had more than two diagnoses. In-hospital mortality was 16%. A missed diagnosis in the emergency department was noted in 101 (20%) patients. The accuracy of the diagnosis of the emergency physician ranged from 0.76 for cardiogenic pulmonary edema to 0.96 for asthma. An inappropriate treatment occurred in 162 (32%) patients, and lead to a higher mortality (25% versus 11%; *p *< 0.001). In a multivariate analysis, inappropriate initial treatment (odds ratio 2.83, *p *< 0.002), hypercapnia > 45 mmHg (odds ratio 2.79, *p *< 0.004), clearance of creatinine < 50 ml minute^-1 ^(odds ratio 2.37, *p *< 0.013), elevated NT-pro-B-type natriuretic peptide or B-type natriuretic peptide (odds ratio 2.06, *p *< 0.046), and clinical signs of acute ventilatory failure (odds ratio 1.98, *p *< 0.047) were predictive of death.

**Conclusion:**

Inappropriate initial treatment in the emergency room was associated with increased mortality in elderly patients with ARF.

## Introduction

In Western countries the population is getting older, and it is projected that the number of people between the age of 65 and 80 years will double by the year 2030 [1,2]. It is estimated that more than 10% of the population over the age of 80 years have heart failure [1]. Acute respiratory failure (ARF) is one of the major causes of consultation of elderly patients in emergency departments (EDs) and is the key symptom of most cardiac and respiratory diseases, such as cardiogenic pulmonary edema (CPE), and of exacerbation of chronic respiratory disease (CRD) including chronic obstructive pulmonary disease (COPD), community-acquired pneumonia (CAP) and pulmonary embolism (PE), which are associated with a high morbidity and mortality [3-8]. In elderly patients, differentiating CPE from respiratory causes is difficult for several reasons. Cardiac and respiratory diseases frequently coexist. Atypical clinical presentation, such as wheezing in CPE (cardiac asthma) or lack of infectious signs in pneumonia, is confusing [5,7,8]. In the oldest patients, autopsy studies have demonstrated that the main causes of death were CPE, CAP, and PE, which are frequently underestimated [9]. There is little knowledge of the presentation, clinical characteristics and outcomes of ARF in elderly patients. Furthermore, two studies suggested that prognosis was improved when early diagnostic and treatments were accurate [10,11].

The objectives of this study were therefore to determine the causes of ARF in elderly patients, the accuracy of the initial diagnosis suspected by the emergency physician, the impact of initial diagnosis and treatment, and variables associated with in-hospital death.

## Methods

### Study design and setting

This epidemiological study of ARF in elderly patients was a single-center prospective study performed from February 2001 to September 2002. It took place in the ED of an urban teaching hospital (2,000 beds), in whom contrast-enhanced helicoidal computed tomography (CT) scan and ultrasonography are available 24 hours a day. Conversely, Doppler echocardiography and other investigations (such as pulmonary function tests (PFTs) or lung scintigraphy) are not easily available in our ED. There is no cardiologist or pulmonologist assessment in the emergency room. During the study period, 90,547 patients have consulted in our ED, of whom 10,156 (11%) were aged more than 65 years. This study was approved by our Ethical Committee, and waived informed consent was authorized because routine care of the patient was not modified.

### Patients

The criteria for inclusion in the study were the following: emergency admission to our ED; age at least 65 years; acute dyspnea of less than two weeks' duration, a subjective criterion defined by the patient (the dyspnea was present if the patient answered one of the following questions in the affirmative: Are you breathless? Do you feel short of breath? Do you experience air hunger? Do you feel increased effort of breathing?); and one of the following objective criteria of ARF: a respiratory rate at least 25 minute^-1^, an arterial partial pressure of oxygen (PaO_2_) of 70 mmHg or less, a peripheral oxygen saturation (SpO_2_) of 92% or less while breathing room air, and an arterial partial pressure of CO_2 _(PaCO_2_) of 45 mmHg or more with an arterial pH of 7.35 or less. There were no exclusion criteria.

### Routine clinical assessment

For every patient, standard medical care provided by the emergency physician (resident or senior) in charge included the following: medical history, physical examination findings including signs of acute ventilatory failure (use of accessory respiratory muscles, paradoxical abdominal respiration), arterial blood gas analysis while breathing room air, 12-lead electrocardiogram, chest X-ray and the usual blood tests. Creatinine levels were measured, and creatinine clearance was estimated with the Cockcroft formula. Dependence and quality of life were assessed by the activity of daily living score [12]. As usual, after the initial presentation in the emergency room, all the patients were reviewed with one of the senior staff members. Thus, depending on the suspected diagnoses, emergency treatment and admission were decided by the emergency physician in accordance with normal practice and recommendations [4,7,13,14]. Usually, dyspneic patients stay for less than eight hours in the emergency room before their admission into another ward.

In accordance with standard practice in our institution for ARF in elderly patients, the performance of thoracic high-resolution computed tomography (HRCT) without contrast iodine medium, of transthoracic Doppler echocardiography with emphasis on diastolic function, and of PFTs was encouraged whenever possible and as quickly as possible during hospitalization. We did not conduct all examinations on every patient because the emergency physician decided whether they were appropriate or not. Almost all thoracic HRCTs were performed less than 12 hours after admission. Slices of 1 mm every 30 mm were performed on inspiration with lung and soft kernels, and read on lung and mediastinal windows respectively. All the CT scans were interpreted again by a radiologist (CB) blinded for any clinical information and medical chart (especially the results of echocardiography and PFTs), and only this interpretation was taken into account by the panel of experts. Transthoracic Doppler echocardiography included two-dimensional and M-mode examination, pulsed Doppler analysis of mitral flow, and continuous Doppler analysis of tricuspid regurgitation. Systolic pulmonary arterial pressure was calculated from the velocity of tricuspid or pulmonary regurgitation, when present. The left ventricular ejection fraction was estimated by visual inspection. PFTs included the measurement of lung volumes and flow-volume loop. In some cases, spirometry was performed at the bedside (*n *= 14) with a validated portable spirometer (Easyone™; Dyn'air, Muret, France) [15]. All PFTs were interpreted again by a pulmonologist (MHB) blinded for any clinical information and medical chart, and only this interpretation was taken account by the panel of experts. Results from various other investigations decided by the physician in charge (in the emergency room or in another medical ward) were also recorded, such as ultrasonography of the lower limbs, contrast-enhanced thoracic CT scan, pulmonary arterial catheter in intensive care unit (ICU), and other relevant results. During this study, levels of B-type natriuretic peptide (BNP) (Triage BNP; Biosite, San Diego, CA, USA) and NT-proBNP (Elecsys 2010 analyse; Roche Diagnostics, Meylan, France) were evaluated in separate studies [16], and the emergency physicians were not aware of the results. As we demonstrated previously in an elderly population, the cutoff values of 250 pg ml^-1 ^for BNP and 1,500 pg ml^-1 ^for NT-proBNP were used in the analysis [16]. The length of hospitalization, admission to the ICU in the first 24 hours, the number of hospital-free days within one month after admission, and the in-hospital mortality during a short stay were also recorded. The final diagnosis of ARF was then determined by two independent senior experts (pulmonologist, cardiologist, general-medicine internist, intensivist, geriatric or emergency physician) from an examination of the complete medical chart including all initial clinical findings, emergency laboratory tests, X-ray chest data, and the results of thoracic HRCT, transthoracic Doppler echocardiography, PFT (or bedside spirometry), and BNP and/or NT-proBNP levels when available. In cases of disagreement between the two experts, a consensus was reached by a third expert. The main final proposed diagnoses were CPE including left heart failure, CAP, acute exacerbation of CRD, PE, acute asthma, bronchitis, and other main diagnoses not listed above, and lack of any diagnosis. The use of validated criteria, response to diuretic or vasodilator, results of echocardiography Doppler, and BNP and NT-proBNP levels performed at admission in the emergency room, and other cardiac tests were specifically analyzed for CPE [5,13,17]. Results from PFT or bedside spirometry, thoracic HRCT and response to bronchodilator or steroids or antibiotics were specifically analyzed for respiratory disorders [7,18]. Results of ultrasonography, contrast-enhanced helical CT scan and perfusion/ventilation nuclear lung scan were specifically analyzed for PE, as recommended [14].

Emergency physicians were asked for their diagnosis just before the patient was leaving the emergency room for either the observation unit of our ED or another ward, including the ICU. According to the final diagnosis made by the experts, an inaccurate emergency physician diagnosis was recorded only if one of the following diagnoses was missed: PE, CPE, CAP, and acute asthma. This was because all these causes of ARF are thought to be linked to increased mortality when initial early treatment is inappropriate, thus requiring early diagnosis. The emergency diagnosis was recorded before thoracic CT scan, Doppler echocardiography or PFT was performed.

The initial specific treatment was defined as that administered when the patient was still in the emergency room. In the same manner, an inaccurate initial treatment was recorded when PE was diagnosed by the experts without initial anticoagulation, when CPE was diagnosed without initial administration of nitrate and/or diuretics, when CAP was diagnosed without initial antibiotics, and when acute asthma was diagnosed without β_2_-agonist administration. Oxygen was considered as a symptomatic treatment not a specific treatment (for example, initial anticoagulation for PE). When patients had two or more causes of ARF, they required treatment for both (for example, diuresis and antibiotics for a patient with congestive heart failure and acute pneumonia) to be considered appropriate.

At the time of the study, non-invasive ventilation or continuous positive airway pressure was not performed in the elderly in the ED, but they were performed in the ICU when the patients were transferred.

### Statistical analysis

The data were collected by three research assistants and verified by one of us (PR). Consistency checks between data entered in the database and searches for erroneous values were performed by one of us (BR) before closing the database and performing statistical analysis. Data are expressed as means ± SD or medians and 95% confidence intervals (CIs) for non-Gaussian variables (Kolmogorov-Smirnov test). Comparison of two means was performed with Student's *t *test, comparison of two medians with the Mann-Whitney test, and comparison of two proportions with Fisher's exact method.

Assessment of the diagnostic performances of the emergency physicians was performed by calculating the sensitivity, specificity, positive and negative predictive values, and accuracy (defined as the sum of concordant cells divided by the sum of all cells in the 2 × 2 table) and their 95% CIs were calculated. The reference diagnosis was that of the experts.

To enable us to compare the length of hospitalization in a short stay in different groups while taking mortality into account, we calculated the number of hospital-free days within one month after admission, as reported previously [19]. Because some of our elderly patients were sent to chronic care before returning home, we considered only hospitalization into an acute care setting for this calculation, and all dead patients were scored 0 hospital-free days.

We performed a multivariate analysis to assess variables associated with missed diagnosis. We also performed a multiple backward logistic regression to assess variables associated with death. To avoid overfitting, we used a conservative approach and included only the significant preoperative variables in the univariate analysis (*p *value of entry < 0.10), except for some variables that were thought to be prognostic or had been demonstrated to be prognostic in previous studies (MacCabe score 3 (death expected in one year) elevated troponin value, elevated natriuretic peptides and clinical signs of acute ventilatory failure). Interactions were not tested. The receiver operating characteristic (ROC) curve was used to determine the best threshold for continuous variables to predict death. The best threshold was the one that minimized the distance to the ideal point (sensitivity = specificity = 1) on the ROC curve. The Spearman coefficient matrix correlation was used to identify significant collinearity (more than 0.70) between variables. Odds ratios and their 95% confidence interval of variables selected by the logistic model were calculated. The discrimination of the model was assessed with the ROC curve and the calculation of the area under the ROC curve [20]. The percentage of patients correctly classified by the logistic model was calculated by using the best threshold determined by the ROC curve. Calibration of the model was assessed with Hosmer-Lemeshow statistics [21].

All statistical tests were two-sided, and *p *< 0.05 was required to reject the null hypothesis. Statistical analysis was performed with NCSS 2001 software (Statistical Solutions Ltd, Cork, Ireland).

## Results

We included 514 patients with ARF; their main characteristics are summarized in Table 1. In our ED, the prevalence of acute dyspnea in patients older than 65 years who consulted at our ED was 5%. All the patients experienced an ED visit and acute dyspnea, and 80% of them had a respiratory rate of at least 25 minute^-1^. Table 2 summarizes the additional inclusion criteria and the clinical severity criteria. Previous medications included diuretics in 167 patients (33%), nitrates in 127 (25%), calcium channel blockers in 130 (25%), inhaled bronchodilatators in 120 (23%), beta-blockers in 72 (14%), angiotensin-converting enzyme inhibitors in 136 (26%), anticoagulation in 53 (10%), and oral corticosteroids in 21 (4%); 23 patients (4%) received home oxygen. A cough was noted in 222 patients (43%), expectoration in 91 (18%), chest pain in 33 (6%), and hemoptysis in 6 (1%) patients. Most of the patients had a normal Glasgow coma scale (*n *= 481 (94%)). The mean serum creatinine was 110 ± 75 μmol l^-1 ^(*n *= 484), with an estimated creatinine clearance of 52 ± 24 ml minute^-1^, 239 of 484 patients (49%) had an estimated creatinine clearance of 50 ml minute^-1 ^or less. An elevated troponin Ic level was observed in 83 of 356 patients (23%).

Thoracic HRCT was performed in 275 patients (54%). Transthoracic Doppler echocardiography was performed in 230 patients (45%), and was performed in 152 of the 212 patients with final diagnostic of CPE PFTs or spirometry was performed in 180 patients (35%), of whom 164 were patients with exacerbation of CRD. Measurements of NT-proBNP or BNP levels were performed in 375 patients (73%).

There was good agreement between experts for the diagnosis of CPE (82%), CAP (87%), PE (91%), acute exacerbation of CRD (83%), and acute asthma (98%). The final diagnoses defined by the experts and their mortality are reported in Table 3; 244 patients (47%) had more than two diagnoses of ARF. Other diagnoses included severe sepsis other than acute pneumonia (*n *= 30), malignancy (*n *= 15), pleural effusion (*n *= 11), tense ascitis (*n *= 5), pneumothorax (*n *= 4), neurologic diseases (*n *= 2), and various other medical diagnoses (*n *= 11). The main causes of CPE that were clearly determined were concomitant exacerbation of COPD in 55 cases, rapid atrial fibrillation in 43, acute coronary syndrome (including 2 with ST-segment elevated myocardial infarction) in 40, CAP in 38, anemia in 20, and associated PE in 11.

All patients except two were admitted to the hospital; 289 patients (56%) were initially admitted to the observation unit of our ED before they were hospitalized in a medical ward, and 74 were admitted directly to a medical ward (14%). During the first 24 hours, 151 patients were admitted to an ICU (29%). The median length of stay in the hospital was 12 days (95% CI 11 to 13) (range 0 to 87). Eighty patients (16%) died in hospital (95% CI 13 to 19). The mortality of patients with two or more final diagnoses was not significantly different from that of patients with only one final diagnosis (18% versus 14%, *p *= 0.11).

**Figure 1:**
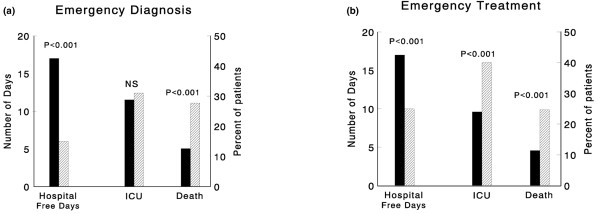
Effects of an appropriate medical care in the emergency department on prognosis. Effects of an appropriate (full bars) or inappropriate (hatched bars) diagnosis in the emergency department **(a) **or initial emergency treatment **(b) **on the number of hospital-free days within 1 month after admission (expressed as median), percentage of patients admitted to intensive care unit (ICU), or mortality. NS, not significant.

The diagnostic performance of the emergency physicians is reported in 4. A missed diagnosis of CPE (*n *= 56), CAP (*n *= 26), PE (*n *= 23) or asthma (*n *= 5) in the ED was noted in 101 (20%) patients. The number of hospital-free days within one month after admission was significantly lower and mortality was significantly higher in patients with a missed diagnosis (Figure 1a). Patients with a missed diagnosis were not different from patients with appropriate diagnosis performed in the emergency ward, in terms of medical past history, clinical signs, and results of laboratory tests. In the multivariate analysis, three variables were significantly associated with a missed diagnosis: final diagnosis of CPE, final diagnosis of PE, and final diagnosis of CAP (5). Conversely, systemic arterial hypertension was associated with fewer missed diagnoses. The Hosmer-Lemeshow statistic was 7.38 (*p *= 0.39), indicating appropriate calibration. The area under the ROC curve was 0.775 ± 0.027 (*p *< 0.05); the accuracy of the logistic model was 0.80 (95% CI 0.72 to 0.82). BNP and NT-proBNP levels were significantly higher in the group of patients with a missed diagnosis, namely 143 pgml^-1 ^(95% CI 101 to 171) versus 403 pg ml^-1 ^(95% CI 337 to 503; *p *< 0.001) and 1,181 pg ml^-1 ^(95% CI 781 to 1,700) versus 3,555 pg ml^-1 ^(95% CI 1,735 to 6,330; *p *= 0.01), respectively.

**Figure 2:**
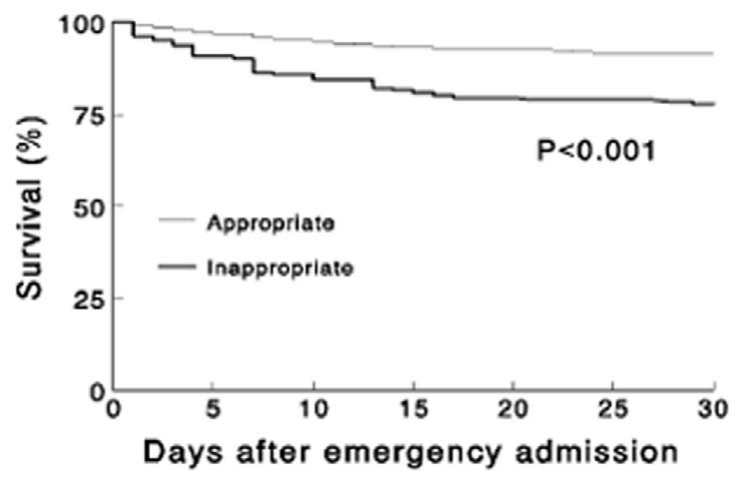
Kaplan-Meier estimates of survival according to the initial treatment received in the emergency department. Inappropriate treatment was noted in 162 (32%) of the 514 patients. The log-rank test was used to calculate *p*.

**Figure 3:**
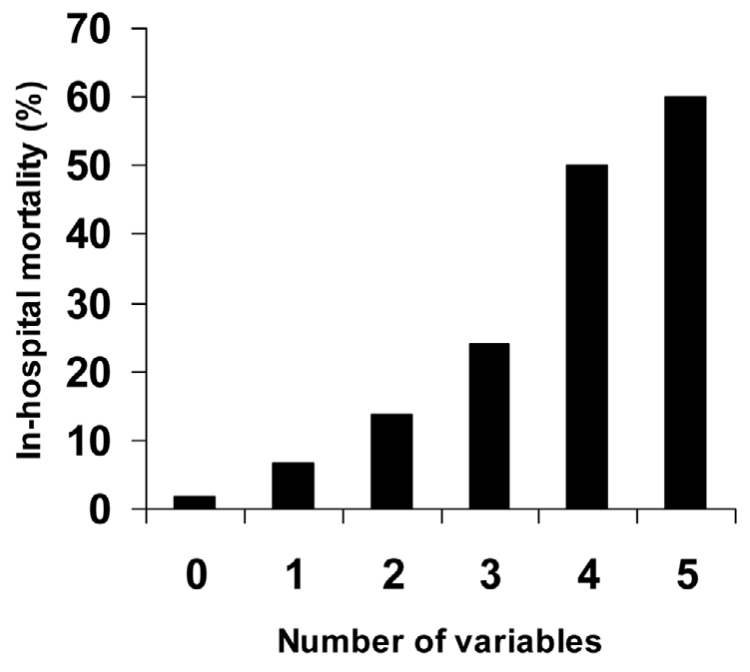
Mortality (%) according to the five variables (X axis) associated with death in the multivariate analysis.

Oxygen was given in 444 patients (86%) in the ED, but was considered a symptomatic rather than a specific treatment. Inappropriate initial treatment of CPE (*n *= 93), CAP (*n *= 56), PE (*n *= 36), or asthma (*n *= 0) was noted in 162 patients (32%). Seventy-nine patients (15%) with a missed diagnosis had also an inappropriate treatment in the emergency room. The number of hospital-free days within one month after admission was significantly lower, and the rates of admission into ICU and mortality (25% versus 11%, *p *< 0.001) were significantly higher, in patients with an inappropriate initial treatment (Figure 1b). Most of the difference in mortality occurred within few days after admission (Figure 2). The following variables were considered in the logistic model to predict death: previous cancer, McCabe score less than one year, any previous cardiac disease, hypotension (systolic arterial pressure less than 90 mmHg), acidosis (pH less than 7.35), hypercapnia (PaCO_2 _more than 45 mmHg), peripheral hypoxia (SpO_2 _less than 92%), elevated serum creatinine (more than 120 μmol l^-1^), low estimated creatinine clearance (less than 50 ml minute^-1^), elevated troponin Ic, elevated NT-proBMP or BNP, hyperkalemia (more than 5 mmol l^-1^), mottling, ventilatory rate more than 30 minute^-1^, clinical signs of ventilatory failure (the use of accessory respiratory muscles and/or abdominal paradoxical respiration), missed diagnosis, and inappropriate treatment in the emergency room. No significant collinearity was noted between these variables. Missing values were observed for natriuretic peptides values (*n *= 139 (27%)), estimated creatinine clearance (*n *= 30 (6%)), and hypercapnia and acidosis (*n *= 24 (5%)). Thus, the final logistic model was performed in 347 (68%) patients. In the multivariate analysis, five variables were significantly associated with mortality: inappropriate initial treatment in the emergency room, hypercapnia, low estimated clearance of creatinine, elevated NT-proBNP or BNP, and clinical signs of ventilatory failure (6). The Hosmer-Lemeshow statistic was 2.22 (*p *= 0.99), indicating appropriate calibration. The area under the ROC curve was 0.767 ± 0.040 (*p *< 0.05). The best threshold for the probability of death was 0.21 with a sensitivity of 0.68 (95% CI 0.42 to 0.68), a specificity of 0.80 (95% CI 0.75 to 0.84). The accuracy of the logistic model was 0.76 (95% CI 0.71 to 0.80). Figure 3 represents the mortality according to the number of variables previously associated with death in the multivariate analysis. The in-hospital mortality varied from 5% when patients had less than two of these variables (*n *= 151) to 52% when patients had four or more variables associated with death (*n *= 27) (Figure 3).

## Discussion

In this large prospective study, which evaluated ARF in elderly patients, the in-hospital mortality was 16%. We observed that predictive variables of mortality were the following: initial inappropriate treatment, hypercapnia at least 45 mmHg, clearance of creatinine 50 ml minute^-1 ^or less, clinical signs of acute ventilatory failure, and elevated BNP or NT-proBNP levels. Inappropriate initial treatment occurred in one-third of cases, and the in-hospital mortality was double that of patients with appropriate treatment.

Both the incidence and prevalence of heart failure, COPD, CAP, and PE are increasing with age [1,7]. Previous studies have reported that CPE is one of the main causes of hospitalization in elderly patients and has a high mortality rate [1,4-6,22]. This was confirmed in our study. However, we also showed that almost half of the patients had more than two causes of ARF. Landahl and colleagues [23] reported that the main causes of dyspnea in 70-year-old people were heart failure, bronchitis, and emphysema. However, this phone-call study evaluated stable dyspnea. In a recent study that evaluated the usefulness of BNP in acute dyspnea, heart failure was the main cause, followed by exacerbation of COPD [10]. All studies that evaluated the accuracy of physical examination in diagnosing causes of dyspnea have also demonstrated that CPE, acute exacerbation of CRD, or respiratory infection were the primary causes of dyspnea [24-30]. In contrast with a younger population, the rate of acute asthma and bronchitis were low [24]. Nevertheless it should noted that all these previous studies had several differences from our study or various biases, namely: various settings (ED, pulmonary clinic, community cohort population sample); middle aged population studied; the number of patients; the numbers of patients excluded from the analysis; the absence of standardized methodology to determine the final diagnosis of acute dyspnea; low mortality; difference in inclusion criteria between isolated dyspnea and ARF; and no emphasis on risk factors of deaths.

Our study demonstrated an in-hospital mortality of 16% (95% CI 13 to 19), with a higher mortality in patients with CPE (21%). These results are similar to previous studies that demonstrated a mortality of severe CPE from 13 to 29% [10,22,31,32]. In a multivariate analysis, we observed that three predictive variables of mortality were easily evaluated in emergency room: hypercapnia at least 45 mmHg, clearance of creatinine 50 ml minute^-1 ^or less, and clinical signs of acute ventilatory failure. Thus, physicians should focus more on these criteria to evaluate the severity of illness in elderly patients with ARF. We also confirmed that elevated BNP or NT-proBNP levels should also be considered prognostic variables [16,33]. Thus, their measurement should be developed in the emergency room, because rapid measurement of BNP in the ED improved the evaluation and treatment of patients with dyspnea, especially in the elderly [10,32].

In our study, the sensitivity of the diagnostic performance of the emergency physician varied from 0.67 for acute asthma to 0.86 for CAP, and the accuracy of the diagnosis of CPE (0.76) was quite similar to that in another study [33]. Several reasons explain the difficulties in assessing causes of ARF in elderly patients. For CPE, atypical presenting symptoms are frequent, such as cardiac asthma presenting as obstructive airways disease or fatigue or leg swelling [5]. Furthermore, classical radiological signs of CPE are sometimes confusing. In cases of CAP, up to 50% of elderly patients had attenuated respiratory symptoms and non-respiratory symptoms such as confusion or falls, and over one-third had no systemic signs of infection [7,8].

We wished to estimate the diagnostic accuracy of the emergency physician and to test whether missed diagnosis and/or inappropriate treatment could be of any prognostic value. In our study three variables were significantly associated with missed diagnosis: a final diagnosis of CPE, PE, or CAP. This result confirms a previous autopsy study [9] that analyzed the clinical and autopsy records of 234 elderly patients: the most common causes of death included bronchopneumonia (33%), congestive heart failure (15%), and PE (8%). Furthermore, the highest diagnostic error rate was in the underdiagnosis of PE (39% ante-mortem accuracy rate only). Conversely, systemic arterial hypertension was significantly associated with fewer missed diagnoses, suggesting that emergency physicians more frequently evoked the diagnosis of CPE in patients with ARF and known hypertension. A missed diagnosis in the emergency room was noted in 20% of patients, leading to a smaller number of hospital-free days within one month after admission and to higher mortality. Interestingly, age over 75 years, dementia, previous quality of life, fatal disease, and clinical presentation were not significantly associated with missed diagnosis. The higher level of BNP and NT-proBNP in the group of patients with a missed diagnosis is probably explained by the fact that CPE represented more than half of the missed diagnoses. Wuerz and Meador [11] suggested in a retrospective pre-hospital study that mortality was reduced in treated patients with CPE in comparison with untreated patients (odds ratio for survival, 2.51 (95% CI 1.37 to 4.55)). We confirmed that undertreatment of the causes of ARF was associated with higher morbidity and mortality (Figure 1b) with a close odds ratio for improved survival (2.83 (95% CI 1.48 to 5.41), *p *< 0.002). Again, age, sex, previous quality of life, respiratory rate, initial severity of hypoxemia, and admission to ICU were not significantly associated with mortality.

Our study has several limitations. We included patients aged more than 65 years although some geriatric physicians now define elderly patients as being more than 75 years. Nevertheless, the median age was 80 years and most of our patients were aged more than 70 years. Our study was monocentric but included consecutively a large cohort of elderly dyspneic patients. Our results, especially the diagnostic performance of emergency physicians and outcomes, could have been modified if the study had taken place in other medical department (a respiratory unit or ICU, for example) or in another country where the medical care of patient is different, whether with cardiologists and pulmonologists in the assessment of the patients in the ED and hospital admission, or if Doppler echocardiography or natriuretic peptides levels were available 24 hours a day, or if non-invasive ventilation was performed in the ED. As one of the inclusion criteria was acute dyspnea (a subjective symptom), it means that patients should have expressed their shortness of breath, which might have excluded some patients with severe neurological diseases but who had ARF. This might explain the good health-related quality of life and the relatively low rate of institutionalized patients in our study population. Nevertheless, the incidences of neurological diseases and other coexisting non-cardiovascular diseases in our patients were similar to those observed previously [10,21,31,32].

Some argue that ARF is usually identified by a PaO_2 _below 60 mmHg and/or a PaCO_2 _above 45 mmHg (irrespective of the degree of respiratory acidosis), and we agree that these usual criteria are stricter than ours. Thus, we used a different cut off point to select ARF from that which is usually considered, with mild hypoxemia (70 mmHg or less). However, some previous studies have already used other criteria in selecting patients for randomized clinical trials in non-invasive ventilation [22], with the use of clinical inclusion criteria (such as polypnea at least 25 per minute or contraction of the accessory muscles of respiration), not only gas exchange impairment. Moreover, it should be noted that we studied elderly patients in whom the capacity to face a respiratory distress is markedly reduced because of the ageing process and/or frequently associated chronic disease. In fact, we believe that our definition was sufficiently large to encompass all causes of ARF that cannot be reduced to those associated with hypoxemia below 60 mmHg. Furthermore, the rate of patients with clinical severity criteria (73%) and ICU admission (29%) observed in our cohort was relatively high, and the mortality was similar to that of elderly patients requiring treatment in the ICU for CAP or for CPE, indicating that we did indeed select a critically ill population [7,22].

The method used in our study to diagnose the cause of ARF requires comment. As in most EDs, Doppler echocardiography is not immediately available and in any case is rarely performed on elderly patients in clinical practice [5,31,34]. Thus, we encouraged, as soon as possible after hospitalization, the use of several non-invasive investigations including HRCT without contrast iodine medium, Doppler echocardiography, and PFTs. Furthermore, to determine the final diagnosis, the experts also had the results of BNP or NT-proBNP levels performed blind at admission, within the framework of a published study [16]. For an evident ethical reason, these investigations were not performed on all patients. In fact, almost all the patients had one of these investigations. Because the agreement between experts was above 85%, we suggest that the final diagnoses by experts were appropriate. Unfortunately, because it is rarely feasible in our country we did not perform an autopsy, which should be considered as the definitive diagnostic test in deceased patients [9]. In our study, the rate of patients with an initial inappropriate treatment (32%) was higher than the rate of patients with an initial missed diagnosis (14%). It should be noted that we recorded the initial treatment administered during the first hours in the emergency room, whereas the diagnosis of the emergency physician was recorded when the patient left the ED. A noticeable delay occurred between these two records, particularly in patients admitted into our observation unit before being sent to another department, usually within less than 24 hours. Although inappropriate treatment in the ED was the main factor associated with increasing mortality, we cannot demonstrate a link of causality between inappropriate initial treatment and outcomes. Thus, because our study was observational, we can only suggest that early appropriate treatments could improve prognosis, and that further studies are merited to confirm that hypothesis. Nevertheless, a prospective randomized controlled study in elderly patients suggested that rapid measurement of BNP in the ED reduced the time to discharge and the total treatment cost, and seemed to reduce 30-day mortality [32].

## Conclusion

We have demonstrated in a large sample of elderly patients with ARF that mortality of patients with inappropriate treatment in the ED was double that of patients with appropriate treatment. To evaluate the severity of illness of elderly patients with ARF, physicians should focus more on easily available criteria associated with higher mortality: hypercapnia, a creatinine clearance of 50 ml minute^-1 ^or less, elevated levels of BNP or NT-proBNP, and clinical signs of acute ventilatory failure. As the size of the geriatric population increases, the medical care of elderly patients with ARF needs to be improved.

## Key messages

• 	The mortality of elderly patients with acute respiratory failure was high (16%).

• 	CPE was the main cause of ARF in elderly patients; however, half of the patients had more than two diagnoses.

• 	An inappropriate initial treatment was associated with increased mortality.

• 	To evaluate the severity of illness of elderly patients with ARF, physicians should focus on available criteria: PaCO_2_, creatinine clearance, levels of BNP or NT-proBNP, and clinical signs of acute ventilatory failure.

## Abbreviations

ARF = acute respiratory failure; BNP = B-type natriuretic peptide; CAP = community-acquired pneumonia; CI = confidence interval; COPD = chronic obstructive pulmonary disease; CPE = cardiogenic pulmonary edema; CRD = chronic respiratory disease; CT = computed tomography; ED = emergency department; HRCT = high-resolution computed tomography; ICU = intensive care unit; PaCO_2 _= arterial partial pressure of CO_2_; PaO_2 _= arterial partial pressure of oxygen; PE = pulmonary embolism; PFT = pulmonary function test; ROC = receiver operating characteristic; SpO_2 _= peripheral oxygen saturation.

## Competing interests

The authors declare that they have no competing interests. The manufacturers (Biosite and Roche Diagnostics) provided the diagnostic tests free of charge.

## Authors' contributions

PR, SB, YL and JB were responsible for study concept and design, acquisition of subjects and/or data, interpretation of data, and preparation of the manuscript.

M-HB, MA, CB, AT, and RI were responsible for acquisition of subjects and/or data, and interpretation of data. BR was responsible for statistical analysis and interpretation of data, and preparation of the manuscript. All authors read and approved the final manuscript.

## Figures and Tables

**Table 1:** Patient characteristics

Variable	Men (*n *= 253)	Women (*n *= 261)	All patients (*n *= 514)
Age (years)	78 ± 8	82 ± 9^a^	80 ± 9
Age ≥ 70 years	157 (62)	201 (77)^a^	358 (70)
Weight (kg)	72 ± 17	62 ± 19^a^	67 ± 9
Body mass index (m^2^.kg^-1^)	25 ± 6	25 ± 8	25 ± 7
Living in an institution	19 (8)	26 (10)	45 (9)
Activity of daily living score	6 [6-6]	6 [6-6]	6 [6-6]
Medical history			
Previous cardiac disease	107 (42)	123 (47)	230 (45)
Coronary artery disease	62	58	120
Valvular disease	13	11	24
Others	38	55	93
Previous cardiac insufficiency	53 (21)	57 (22)	110 (21)
Hypertension	122 (48)	151 (58)^a^	275 (53)
Chronic respiratory disease	93 (37)	40 (15)^a^	133 (26)
Obstructive	84	13	97
Restrictive	3	6	9
Mixed or others	6	21	27
COPD	104 (41)	21 (8)^a^	125 (24)
Asthma	9 (4)	21 (8)^a^	30 (6)
Diabetes	47 (19)	34 (13)	81 (16)
Chronic renal insufficiency	7 (3)	9 (3)	16 (3)
Cancer	48 (19)	28 (11)^a^	76 (15)
Remission	16	14	30
Stable	7	3	10
In progress	25	11	36
MacCabe score 3	113 (45)	63 (24)^a^	176 (34)
Previous neurological disease	46 (18)	44 (17)	90 (18)
Cerebrovascular disease	18	15	33
Parkinson's disease	9	15	24
Dementia	11	10	21
Others	8	4	12
Clinical signs			
Respiratory rate (minute^-1^)	27 ± 6	29 ± 6^a^	28 ± 6
Cardiac rate (minute^-1^)	94 ± 20	92 ± 20	93 ± 20
Systolic arterial pressure (mmHg)	140 ± 30	145 ± 32^a^	142 + 31
Diastolic arterial pressure (mmHg)	76 ± 17	76 ± 19	76 + 18
Temperature (°C)	37.5 ± 1.0	37.3 ± 1.2	37.4 ± 1.1
Blood gas analysis (*n *= 490)			
pH	7.43 ± 0.07	7.43 ± 0.08	7.43 ± 0.07
PaO_2 _(mmHg)	65 ± 18	65 ± 21	65 ± 20
PaCO_2 _(mmHg)	41 ± 12	41 ± 14	41 ± 13
Bicarbonates (mmol l^-1^)	26.7 ± 5.3	26.7 ± 5.7	26.7 ± 5.5

Data are means ± SD, or number (%). ^a^*p *< 0.05 compared with men. COPD, chronic obstructive pulmonary disease; PaO_2_, arterial partial pressure of oxygen; PaCO_2_, arterial partial pressure of CO_2_; Mac Cabe score 3 : death expected in 1 year.

**Table 2:** Additional inclusion criteria and severity of acute respiratory failure

Criterion	Number of patients (%), or value (mean ± SD)	Number of additional criteria	Number of patients (%)
Additional			
Ventilatory rate ≥ 25 minute^-1^	413 (80)	None	0
Ventilatory rate (mm^-1^)	30 ± 6	1 criterion	162 (31)
PaO_2 _≤ 70 mmHg	342 (67)	2 criteria	189 (37)
PaO_2 _(mmHg)	57 ± 9	3 or more	163 (32)
PaCO_2≥ _45 mmHg and pH ≤ 7.35	44 (9)		
PaCO_2 _(mmHg)	67 ± 19		
pH	7.28 ± 0.09		
SpO_2 _≤ 92%	241 (47)		
SpO_2 _(%)	83 ± 9		

Criteria of clinical severity		Number of criteria of clinical severity	Number of patients (%)

Heart rate ≥ 120 minute^-1^	52 (10)	None	139 (27)
Heart rate (mm^-1^)	129 ± 14	1 criterion	114 (22)
Orthopnea	255 (50)	2 criteria	89 (17)
Abdominal respiration	77 (15)	3 or more	172 (34)
Use of accessory muscles	146 (28)		
Cyanosis	56 (11)		
Ventilatory rate ≥ 30 minute^-1^	100 (19)		
Encephalopathy	13 (3)		
Mottling	37 (7)		
Clinical signs of right heart failure^a^	288 (56)		

All patients fulfilled the other inclusion criteria (namely admission to the emergency department, acute dyspnea, and age ≥ 65 years). Data are number (%) and means ± SD for the numeric variables in patients who fulfilled the given criteria. ^a^Abdominal jugular reflux and/or jugular venous pulse. PaO_2_, arterial partial pressure of oxygen; PaCO_2_, arterial partial pressure of CO_2_; SpO_2_, peripheral oxygen saturation.

**Table 3:** Diagnosis of causes of acute respiratory failure by experts, and mortality

Diagnosis	Number of patients (%)	Mortality^a^, %
Cardiogenic Pulmonary Edema	219 (43)	21 [16–27]
Community-acquired pneumonia	181 (35)	17 [12–23]
Exacerbation of chronic respiratory disease	164 (32)	12 [8–18]
Pulmonary embolism	93 (18)	15 [9–24]
Bronchitis	23 (4)	4 [0–21]
Acute asthma	15 (3)	0 [0–20]
Others	78 (15)	24 [16–34]
No diagnosis	8 (2)	0 [0–32]

Ranges in square brackets are 95% confidence intervals. Because several causes could occur in the same patient, the percentages do not total 100%. ^a^Percentages represent mortality in each diagnostic category.

**Table 4:** Assessment of the diagnostic performance of the emergency physicians (*n *= 514)

Diagnosis	Sensitivity	Specificity	Positive predictive value	Negative predictive value	Accuracy
CPE	0.71 [0.65–0.77]	0.80 [0.75–0.84]	0.74 [0.70–0.87]	0.78 [0.72–0.82]	0.76 [0.72–0.80]
CAP	0.86 [0.80–0.90]^a^	0.76 [0.71–0.80]	0.66 [0.59–0.71]^a^	0.91 [0.87–0.93]^a^	0.79 [0.75–0.82]
Acute exacerbation of CRD	0.71 [0.64–0.78]	0.83 [0.79–0.87]	0.66 [0.59–0.73]^a^	0.86 [0.82–0.89]^a^	0.81 [0.78–0.84]^a^
Pulmonary embolism	0.75 [0.66–0.83]	0.78 [0.74–0.82]	0.43 [0.36–0.51]^a^	0.93 [0.90–0.96]^a^	0.78 [0.74–0.81]
Asthma	0.67 [0.42–0.85]	0.97 [0.95–0.98]^a^	0.42 [0.24–0.61]^a^	0.99 [0.98–1.00]^a^	0.96 [0.94–0.98]^a^

Data are value [95% confidence interval]; ^a^*p *< 0.05 compared with CPE. CPE, cardiogenic pulmonary edema; CAP, community-acquired pneumonia; CRD, chronic respiratory disease.

**Table 5:** Variables associated with missed diagnosis in the emergency department

Variable	Appropriate diagnosis (*n *= 413)	Missed diagnosis (*n *= 101)	Adjusted odds ratio	*p*
History of arterial hypertension	288 (55)	45 (45)	0.59 [0.36–0.98]	0.04
Final diagnosis of CAP	152 (37)	67 (66)	4.85 [2.73–8.61]	<0.001
Final diagnosis of CPE	135 (33)	46 (46)	9.35 [5.16–16.14]	<0.001
Final diagnosis of PE	60 (15)	33 (33)	9.27 [4.72–18.22]	<0.001

Data are numbers of patients (%) or medians [95% confidence intervals]. All differences between survivors and dead patients in the univariate analysis were significant (*p *< 0.05). CAP, community-acquired pneumonia; CPE, cardiogenic pulmonary edema; PE, pulmonary embolism.

**Table 6:** Variables associated with in-hospital death

Variables	Alive (*n *= 434)	Dead (*n *= 80)	Adjusted odds ratio	*p*
Inappropriate treatment in the ED	73/434 (17)	28/80 (35)	2.83 [1.48–5.41]	0.002
PaCO_2 _≥ 45 mmHg	90/414 (21)	29/73 (40)		0.004
PaCO_2 _(mmHg)	41 ± 13 (*n *= 414)	44 ± 14 (*n *= 73)	2.79 [1.39–5.58]	
Creatinine clearance ≤ 50 ml minute^-1^	191/410 (47)	48/74 (65)		0.013
Creatinine clearance (ml minute^-1^)	54 ± 26 (*n *= 410)	43 ± 18 (*n *= 74)	2.37 [1.20–4.71]	
Elevated natriuretic peptide	148/317 (47)	43/58 (74)	2.06 [1.01–4.18]	0.046
BNP (pg ml^-1^)	148 [102–178] (*n *= 275)	371 [237–503] (*n *= 54)		
ProBNP (pg ml^-1^)	1,172 [748–1,700] (*n *= 208)	4,084 [1,317–7,887] (*n *= 38)		
Clinical signs of acute ventilatory failure	91/434 (21)	36/80 (45)	1.98 [1.01–3.90]	0.047

Data are numbers/totals of patients (%), means ± SD for the numeric variables in patients who fulfilled the given criteria, or medians [95% confidence intervals]. All differences between survivors and dead patients in the univariate analysis were significant (*p *< 0.05), including continuous variables. The logistic model was finally applied in 347 patients. Clinical signs of acute ventilatory failure included the use of accessory respiratory muscles and abdominal paradoxical respiration. ED, emergency department; PaCO_2_, arterial partial pressure of CO_2_; BNP, B-type natriuretic peptide.
